# Ice-templated synthesis of multifunctional three dimensional graphene/noble metal nanocomposites and their mechanical, electrical, catalytic, and electromagnetic shielding properties

**DOI:** 10.1038/srep17726

**Published:** 2015-12-07

**Authors:** P. K. Sahoo, Radhamanohar Aepuru, Himanshu Sekhar Panda, D. Bahadur

**Affiliations:** 1 IITB-Monash Research Academy, Indian Institute of Technology Bombay, Mumbai-400076, India; 2 Department of Materials Engineering, Defence Institute of Advanced Technology, Pune - 411025, India

## Abstract

*In-situ* homogeneous dispersion of noble metals in three-dimensional graphene sheets is a key tactic for producing macroscopic architecture, which is desirable for practical applications, such as electromagnetic interference shielding and catalyst. We report a one-step greener approach for developing porous architecture of 3D-graphene/noble metal (Pt and Ag) nanocomposite monoliths. The resulting graphene/noble metal nanocomposites exhibit a combination of ultralow density, excellent elasticity, and good electrical conductivity. Moreover, in order to illuminate the advantages of the 3D-graphene/noble metal nanocomposites, their electromagnetic interference (EMI) shielding and electrocatalytic performance are further investigated. The as-synthesized 3D-graphene/noble metal nanocomposites exhibit excellent EMI shielding effectiveness when compared to bare graphene; the effectiveness has an average of 28 dB in the 8.2–12.4 GHz X-band range. In the electro-oxidation of methanol, the 3D-graphene/Pt nanocomposite also exhibits significantly enhanced electrocatalytic performance and stability than compared to reduced graphene oxide/Pt and commercial Pt/C.

Graphene, a two-dimensional structured carbon material, has captured the attention of the scientific community considerably due to its exceptionally high mechanical strength, ultrahigh specific surface area, and unique conductivity^1^,^2^. These properties make it a promising material for potential applications such as energy storage^2^, supercapacitors^1^,^3^ electrocatalysts for fuel cells^4^,^5^,^6^, water splitting^7^, solar cells^8^, and electromagnetic interference (EMI) shielding^9^. Further, in order to meet the requirements of practical applications, current studies have been focused on improving the properties and the performance of graphene by doping it with nitrogen^4^,^5^,^6^, and decorating it with metals^10^,^11^,^12^, metal-oxide^6^ and various composite materials^5^,^13^. A synergistic combination of these foreign materials with graphene sheets enables them to produce composite materials with superior performances in various applications such as energy storage^2^, electrocatalysts for fuel cells^4^,^5^,^6^, catalysis^14^, water splitting^7^, sensors^15^, supercapacitors^16^,^17^, solar cells^18^, high-performance lithium ion batteries^19^, and EMI shielding^20^. In these composite materials, graphene powder (that is, chemically exfoliated graphene oxide (GO) and reduced graphene oxide (RGO))^7^,^13^,^21^,^22^ has been considered as a supporting material. However, the high degree of aggregation and surface defects in GO and RGO results in a significant reduction in the surface area and alters the conductivity. Thus, there is a need to formulate an effective and reliable way to improve the performance of graphene-based supporting materials.

Recently, three dimensional (3D) graphene assemblies have been used as supporting materials for many applications because they possess better properties (increase in conductivity, surface area and mass transport efficiency) when compared to graphene powder^23^. A number of different approaches have been employed for the fabrication of the 3D-graphene assemblies^24^,^25^,^26^,^27^,^28^,^29^. However, the fabrication processes are quite expensive and hazardous to the environment. A major challenge is the fabrication of well-defined meso-micro-porous 3D-graphene structures at a low cost and with eco-friendly processes. Qiu Ling *et al.* have reported ultra-light 3D-graphene–based cellular monoliths using a cost-efficient freeze-casting process with ultra-low density, superelasticity, good electrical conductivity, and high-energy absorption efficiency^30^. Such an uncommon carbon-based cellular material opens up numerous opportunities in an extensive variety of innovative applications. Here, our objective is to fabricate 3D-graphene/noble metal (Pt or Ag) nanocomposites by taking advantage of this 3D-graphene monolith structure for EMI shielding and methanol fuel cell applications.

In the present work, we develop a greener, efficient, and low cost one-step approach to large-scale production of 3D-graphene/noble metal (Pt or Ag) nanocomposites by freeze-casting of partly RGO and metal salt solutions. The as-obtained 3D graphene/noble metal (Pt or Ag) nanocomposites were characterized by various spectroscopy studies and correlated with microscopic and surface area analysis. The structure, electrical conductivity, and mechanical properties of as-synthesized 3D-graphene/noble metal (Pt and Ag) nanocomposites were also investigated. The resulting 3D-graphene/noble metal (Pt and Ag) nanocomposites show a combination of ultralow density, excellent elasticity, and good electrical conductivity. Further, the enhanced conductive 3D-graphene/noble metal (Pt and Ag) nanocomposites were extended to EMI studies, in which enhanced conductivity is a necessary criterian. It is interesting that the 3D-graphene/noble metal (Pt and Ag) nanocomposites showed enhanced shielding effectiveness when compared to bare 3D-graphene. In addition, 3D-graphene/Pt nanocomposite is used in electrocatalytic activity study of methanol electro-oxidation. Here, the large and accessible pores of variable sizes helps for quick transportation of reactants to electro-active sites is provided by the 3D-graphene network; in addition, the high electrical conductivity of catalysts are maintained overall. As a result, in the electro-oxidation of methanol, 3D-graphene/Pt nanocomposites show excellent electrocatalytic properties such as a high electrocatalytic performance, abnormal poison tolerance, and dependable stability, which are of a higher grade compared to those of Pt/reduced graphene oxide nanocomposites and commercial Pt/C.

## Experimental Methods

### Materials

Natural graphite powder of a particle size of 45 μm (99.99% purity), Hexachloroplatinate (H_2_PtCl_6_.6H_2_O), and Silver nitrate (AgNO_3_) were purchased from Sigma-Aldrich. All other chemicals employed were of analytical grade, purchased from Merck Specialties Private Limited, India. Commercial Pt/C (Pt on graphitized carbon, 20 wt%) was obtained from Sigma-Aldrich. All the chemicals employed were used as-received without any further purification.

### Preparation of 3D-graphene and 3D-graphene/noble metal (Pt and Ag) nanocomposites

**Figure 1:**
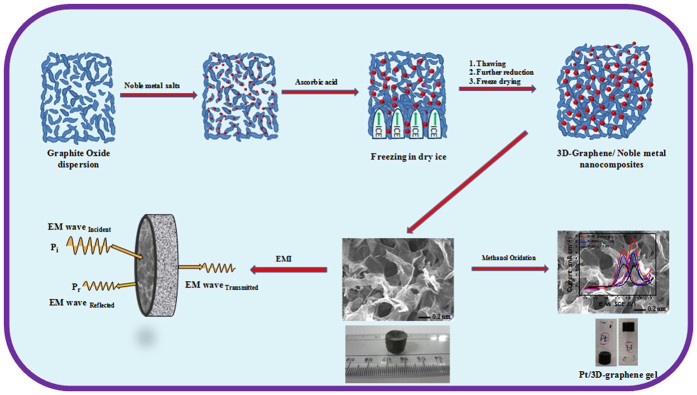
Schematic representation of 3D-graphene/noble metal nanocomposites by the freeze-casting method, and its application in EMI shielding and electrocatalytic oxidation of methanol.

GO aqueous dispersions was prepared by using a reported literature process^30^,^31^, and further exfoliated by using a bath sonicator. In a typical methods for preparing 3D-graphene/noble metal (Pt and Ag) nanocomposites, 1.5 ml of GO solution (5 mg/ml) was blended with respective noble metal salts (H_2_PtCl_6_.6H_2_O = 4.5 mg/AgNO_3_ = 2.5 mg) separately in a cylindrical glass vial of a capacity of 4 ml. To this, ascorbic acid (15 mg) was added and kept in a heating oven at 100 °C for 30 min to get partly reduced GO dispersion and a reduction of metal ions. Each vial was then frozen for 0.5 h in a dry ice bath. After being thawed (at room temperature), the vial was put into a heating oven at 100 °C for 4 h, to further reduce GO to obtain a gel. The gel was then successively subjected to dialysis (to remove dissolved species), freezing drying, and was then dried for 24 h at 50 °C. A detailed schematic representation of the synthesis procedure is presented in the Fig. 1. For comparative study, bare 3D-graphene and RGO/Pt (20 wt%) nanocomposites were synthesized by using ascorbic acid as a reducing agent in the presence and absence of the freeze-casting procedure, respectively.

### Instrumentation and measurements

The structural examination of as-synthesized specimens were performed by using a Powder XRD diffractometer (Philips PW 3040/60) with Cu Kα radiation (*λ* = 1.541 Å); a Magna-IR spectrometer-50 (Nicolet) was used to record FTIR spectra by a standard KBr pellet method. Raman scattering was performed by a RAM HR 800 Micro laser Raman system with a 519 nm Ar^+^ laser. The chemical states and surface compositions of the as-synthesized samples were analyzed using XPS (MULTILAB from Thermo VG Scientific, Mono monochromatic Al Kα X-rays (1486.6 eV). The morphology of the samples was examined by HRTEM using the Phillips-CM 200 electron microscope. The composition of the as-prepared samples was analyzed by an ICP-AES (Prodigy, Teledyne Leeman Labs). The specific surface area, pore volume and size distribution of as-synthesized samples were analyzed by Micromeritics instrument ASAP 2020, and the specific surface areas were determined by the multipoint Brunauer−Emmet−Teller (BET) method. The samples were degassed at 60 °C for 4 h, before the analysis. Cylindrical-shaped (~10 mm in height and ~12 mm in dia.) samples were prepared for electrical conductivity measurements and compressive tests. A broadband dielectric spectrometer (Novocontrol) with an Alpha-A frequency analyzer were used to study the AC conductivity of the as-synthesized samples in the frequency range of 1 Hz to 10 MHz. Conductivity measurements were carried out by putting the samples in between the gold-plated copper electrodes. The compressive tests were executed in an Instron (Micro Tester, 5848) that utilized a load cell (10 N) and had a strain rate of 100% per minute. EMI shielding properties were measured by using PNA Network Analyzer N5222A (Agilent) in the X-band. Catalyst ink was prepared by dispersing 5 mg of each catalyst in a mixed solution of 5 mL of Millipore water, 1 mL of iso-propanol, and 10 μL of Nafion® solution under sonication for 20 min. The 20 μL of the catalyst ink (well dispersed) was drop-casted on the glassy-carbon electrode (0.07 cm^2^), named as the modified glassy-carbon electrode. Potentiostat/Galvanostat Autolab-302N with GPES 4.9 software was used for the electrochemical measurements. Electrochemical measurements were conducted in a three-electrode system. A platinum wire and a saturated calomel electrode (SCE) were used as counter electrode and reference electrode, respectively. A modified glassy-carbon electrode (3 mm diameter) was used as a working electrode. All the measurements were executed at room temperature. The volumetric solution was purged with high purity nitrogen gas for 5–15 min before an electrochemical scan. All the electrochemical potentials are reported against the reversible hydrogen electrode (RHE) in the manuscript.

## Results and Discussion

We demonstrated the fabrication of 3D-graphene/noble metal (Pt and Ag) nanocomposites by using a low cost wet shaping technique, which generally called freeze casting. In this process, phase separation took place between the solid particles and forming ice, and the rejected particles mounted up between the developing ice templates^32^,^33^. The entrapped particles form 3D network when the fraction of particles exceed the percolation limit, and further sublimation of the ice results porous solid monoliths. Recent past, freeze casting method has been widely implemented for preparing variety of porous materials^28^,^30^,^33^,^34^,^35^,^36^,^37^,^38^. In the present work, we have implemented the principle of the freeze-casting process for the formation of 3D-graphene/noble metal (Pt and Ag) nanocomposites. GO and noble metal was chosen together as originator for preparing porous 3D monoliths.

GO and metal salt solution in presence of ascorbic acid produced 3D porous assemblies in freeze-casting process. To understand the mechanism, a control experiment was conducted by using a reported process^30^. It is observed that, the gelation time and annealing temperature are reduced around half and one fourth respectively of the synthesis process in presence of noble metals than bare 3D-graphene monoliths. The chelation of noble metals with unreduced functional group of graphene oxide sheet and water molecules promotes to form 3D gel and such type of phenomenon observed also in GO/noble metal sponge (hydrothermal process) by Tang *et al.* group^25^. Also, the chelation of graphene with noble metals evidenced by FTIR spectroscopy and XPS, and discussed in the later section. In the frozen step, the partially reduced GO sheets chelated with metals are rejected from the forming ice and trapped between neighboring ice templates for forming a continuous network like structure. The 3D-graphene/noble metal complex forced to align with the growth direction of ice crystals. Further, chelation of graphene sheets on noble metals assist the assemble process, and increases the π- π interaction between the graphene layers. The resulting 3D network like structure was found to be quite stable and could maintain its structural integrity upon thawing, reduction, dialysis and freeze-drying processes. Developed materials are characterized by using various sophisticated analytical techniques for exploring the properties and applications.

**Figure 2:**
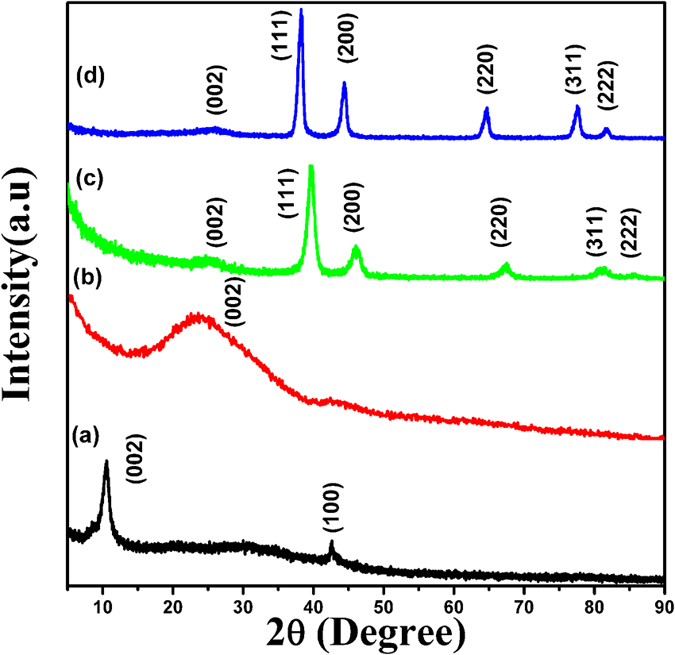
XRD patterns of (**a**) GO, (**b**) bare 3D-graphene, (**c**) 3D-graphene/Pt and (**d**) 3D- graphene/Ag nanocomposites.

XRD was used to explicate the phases and structural parameters of the as-synthesized GO and 3D-graphene/noble metal (Pt and Ag) nanocomposites samples; the results are shown in Fig. 2. In Fig. 2(a), the diffraction peaks at 2θ = 10.8° and 43° correspond to the (002) and (100) plane of graphite oxide and the hexagonal structure of carbon^39^, respectively. However, the peak (2θ = 10.8°) disappeared and reappeared at ~2θ = 24° in the XRD patterns of bare 3D-graphene in Fig. 2(b), indicating that graphite oxide was effectively reduced and there is a existence of π-π stacking between the graphene sheets, which leads to an ordered crystalline structure^40^. As shown in the Fig. 2(c,d), the diffraction patterns of Pt and Ag correspond to the (111), (200), (220), (311) and (222) planes of the fcc structure in the 3D-graphene/Pt and 3D-graphene/Ag nanocomposites. Again, the diffraction peak appeared from the stacked graphene sheets nearly disappeared in the 3D-graphene/Pt and 3D-graphene/Ag nanocomposites, suggested the hindrance of restacking in graphene sheets, which might be due to the attachment of Pt and Ag nanoparticles on the surface of the graphene sheets^41^. The average crystallite size of Pt and Ag in the 3D-graphene/noble metal (Pt and Ag) nanocomposites that was derived from the Scherrer formula is 7 and 16 nm, respectively. Moreover, the XRD pattern of the RGO/Pt nanocomposite is given in Fig. S1 (Supporting information). The diffraction peaks shows fcc structure of Pt (JCPDS 04-0802) in the RGO/Pt nanocomposite.

Raman spectroscopy is extensively used to observe the electronic and structural properties in carbonaceous materials. Figure S2(A) shows the characteristic Raman spectra of GO, bare 3D-graphene and 3D-graphene/noble metal (Pt and Ag) nanocomposites. All spectra showed the existence of characteristic D, G, and 2D bands of graphene/graphene oxide. In case of GO, the G band is centered at 1592 cm^−1^, and shifted to 1577, 1579 and 1580 cm^−1^ for the bare 3D-graphene, 3D-graphene/Pt and 3D-graphene/Ag respectively, which is close to the value of pristine graphene (Fig. S2(B)). The shifting of G band confirmed that the successful reduction of GO in the chemical reduction process^42^. The D band of the GO, bare 3D-graphene, 3D-graphene/Pt and 3D-graphene/Ag nanocomposites is centered at 1355, 1345, 1346 and 1345 cm^−1^ respectively, and predicts the defect and the size of the in-plane sp^2^ domain. A change in the relative intensity of the D and G bands (I_D_/I_G_) is correlated with the change in the average size of the sp^2^ domains. The I_D_/I_G_ ratios for GO, bare 3D-graphene, 3D-garphene/Pt and 3D-graphene/Ag nanocomposites are calculated to be 0.82, 1.1, 1.11 and 1.13 respectively. The increase in I_D_/I_G_ ratios of bare 3D-graphene, 3D-garphene/Pt and 3D-graphene/Ag nanocomposites than GO indicated that the decrease in the average size of the sp^2^ domains. Even, the created new sp^2^ domains of reduced GO are smaller in size than the ones which is present in GO before reduction^43^.

The FTIR spectras of the as-prepared 3D-graphene–based noble metal (Pt and Ag) nanocomposites along with GO are shown in Fig. S3 (supporting information). Figure S3(a) showed the characteristic bands of GO. The characteristic bands of GO are observed at 3400 cm^−1^ (O-H stretching), 1730 cm^−1^ (C=O stretching vibration of the COOH groups), 1405 cm^−1^ (O-H deformation vibration of tertiary C-OH), 1222 cm^−1^ (C-O stretching vibration of phenolic C-OH), and 1045 cm^−1^ (C-O stretching vibrations of epoxy groups). The band at 1612 cm^−1^ is related to the bending vibration of the adsorbed water molecules or skeletal vibrations of unoxidized C-C bonding^44^. Almost all of the characteristic bands of the functional groups disappeared in the FTIR spectra, except C=O stretching vibration of -COOH and O-H stretching and bending vibrations of water in 3D-graphene noble metal (Pt and Ag) nanocomposites. The C=O stretching vibration of GO, 3D-graphene/Pt and 3D-graphene/Ag are measured to be 1730, 1717 and 1725 cm^−1^ respectively. The shifting of the stretching vibration peaks towards lower wave number with increase in mass of the noble metals suggested the chemical interaction of metal ions with the graphene sheets. Also, O-H bending vibration peaks of adsorbed water molecules was become more prominent in 3D-graphene/noble metal nanocomposites and shifted to lower wave number with increasing the mass of noble metals. The O-H bending vibration peaks of H_2_O are observed at 1612, 1568 and 1572 cm^−1^ for GO, 3D-graphene/Pt and 3D-graphene/Ag monoliths respectively. The significant shifting of wave number towards lower side in 3D-graphene/noble metal nanocomposite might be due to the chelation of water molecules with the noble metals.

**Figure 3:**
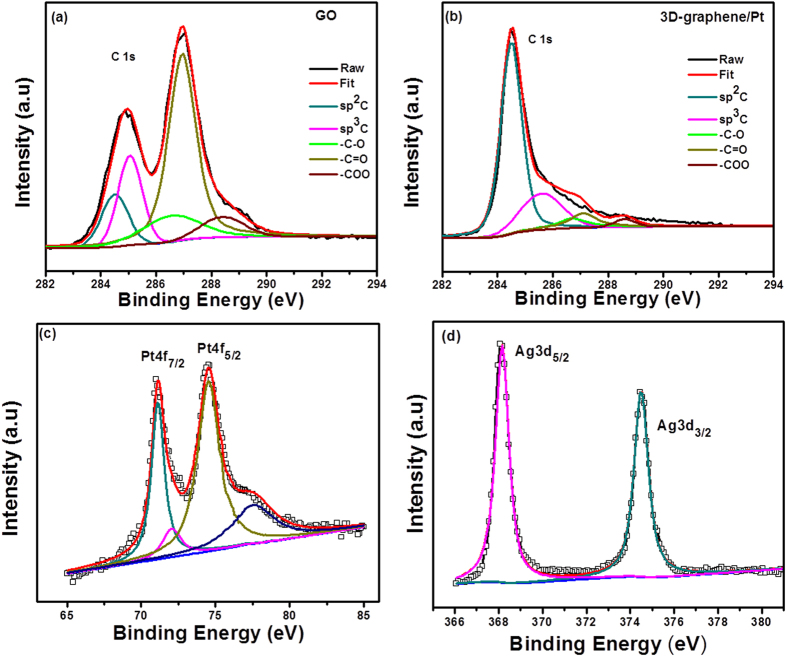
XPS spectra of the (**a**) GO, (**b**) 3D-graphene/Pt, (**c**) Pt 4f_7/2_ and Pt 4f_5/2_ peaks of 3D-graphene/Pt and (**d**) Ag 3d_5/2_ and 3d_3/2_ peaks in 3D-graphene/Ag nanocomposites.

The surface chemistry of the GO, 3D-graphene/Pt and 3D-graphene/Ag nanocomposites was investigated by XPS analysis. Figure 3(a,b) showed the high-resolution spectra in the region of the C 1s characteristic peak of GO and 3D-graphene/Pt nanocomposites. This region showed the different carbon-oxygen binding arrangements. It has been noticed that various oxygenic functional groups (-C-O = 286.6 eV, -C=O = 286.9 eV, -COO = 288.4 eV) are present at the higher binding energy sides of the C1s region for GO^45^. Compared to the intensity of the sp^2^ carbon peak (284.5 eV), the intensities of the various oxygenic functional groups and the intensity of the sp^3^ carbon peak (285.1 eV) were effectively reduced in the case of the 3D-graphene/Pt nanocomposite. It was further confirmed that GO was successfully reduced to graphene in the 3D-graphene/Pt nanocomposite through the freeze-casting method. Figure 3(c,d) presents a high-resolution XPS spectrum of Pt and Ag in the 3D-graphene/Pt and 3D- graphene/Ag nanocomposites, respectively. In Fig. 3(c), the Pt 4f signal is de-convoluted into two components (4f_5/2_ and 4f_7/2_). The most intense doublet Pt 4f peaks at 71.1 eV (4f_7/2_) and 74.6 eV (4f_5/2_ ) are assigned to metallic Pt^0^, whereas the second doublet Pt 4f peaks at 72.1 and 77.51 eV are assigned to Pt^II^. The appearance of Pt^II^ could arise from the partial oxidation of Pt^0^ on the graphene surface. Moreover, from Fig. S4 (Supporting information), it is seen that there is a blue shift of the binding energy of oxygen 1s in the 3D-graphene/Pt nanocomposite than oxygen 1s of the GO. The blue shift of oxygen 1s in 3D-graphene/Pt nanocomposite suggested that the covalent linkage of Pt with sp^2^ carbon of graphene *via* oxygen (Pt-O-C)^46^. In Fig. 3(d), the peaks located at around 368.1 eV and 374.4 eV belong to Ag 3d_5/2_ and Ag 3d_3/2_ respectively, and suggested that the presence of metallic Ag^0^ 47.

**Figure 4:**
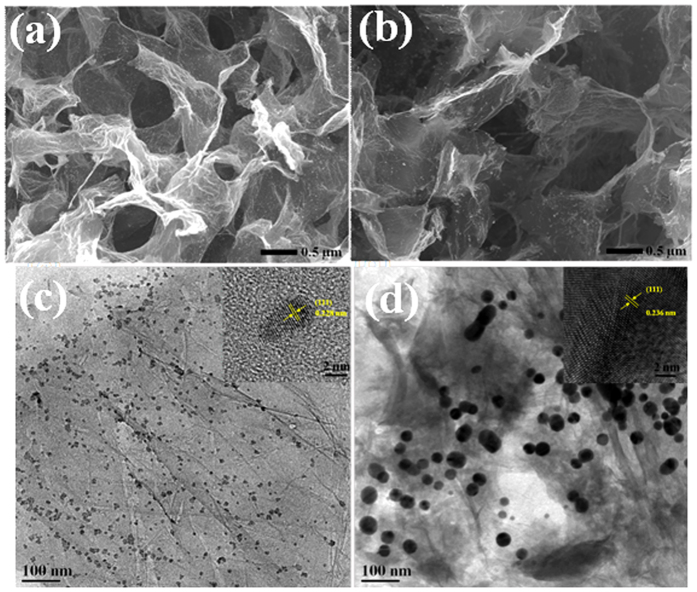
FEG-SEM images of (**a**) 3D-graphene/Pt and (**b**) 3D-graphene/Ag nanocomposites; TEM and HRTEM images of (**c**) 3D-graphene/Pt and (**d**) 3D-graphene/Ag nanocomposites.

The surface morphologies of the 3D-graphene/noble metal nanocomposites were examined by FEG-SEM. Figure 4(a,b) show the 3D porous arrangement with continues open pores that vary from sub-micrometer to several micrometers range. Close inspection reveals that various nanoparticles are homogenously distributed on the surface of the 3D-graphene nanosheets. TEM images of different 3D-graphene/noble metal (Pt and Ag) nanocomposites are presented in Fig. 4(c,d). The noble metal nanoparticles (Pt and Ag) are highly monodispersed and uniformly distributed throughout the surface of graphene sheets. This result indicates that graphene sheets effectively prevent the aggregation of ultrafine metal nanoparticles and confirms a strong interaction between the noble metal nanoparticles and the graphene sheet. The average size of different noble metal nanoparticles are 5 and 13 nm for Pt and Ag, respectively. Before TEM characterization, all the 3D-graphene/noble metal (Pt and Ag) nanocomposites were ultrasonicated for 15 minutes. The noble metal nanoparticles remained attached on the graphene sheets, suggesting that they were strongly anchored on the surface of graphene. The HRTEM images in the inset of Fig. 4(c,d) show that the noble metal nanoparticles (Pt and Ag ) within the graphene sheets exhibit high crystallinity, and the lattice spacings are about 0.228 nm and 0.236 nm, which corresponds to the (111) plane spacing of Pt and Ag crystals, respectively. It is worth mentioning the loading amount of noble metal nanoparticles on the graphene sheets because it has a direct effect on the morphology and the catalytic performance. The loading amounts of different noble metal nanoparticles were analyzed by ICP. It was found that the loading amount of Pt and Ag nanoparticles were 20 percent.

**Figure 5:**
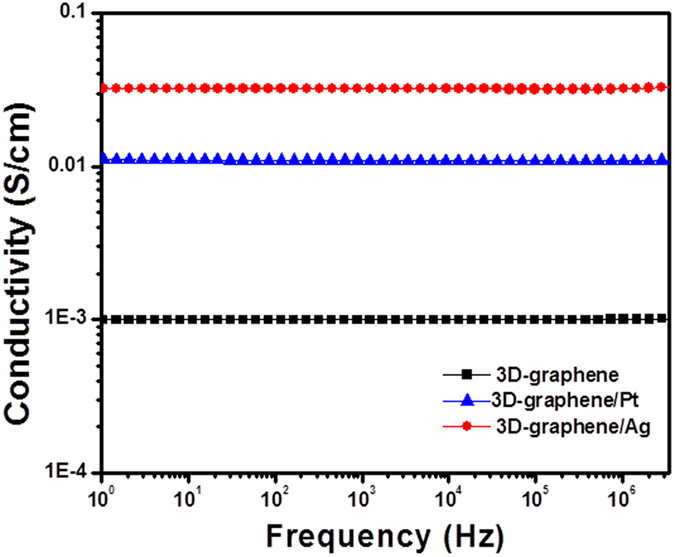
Frequency-dependent real part of AC conductivity of 3D-graphene, 3D-graphene/Pt, and 3D-graphene/Ag nanocomposites.

Figure 5 shows the AC conductivity measurements of bare 3D-graphene and 3D-graphene/noble metal (Pt and Ag) nanocomposites. As indicated, the 3D-graphene/noble metal nanocomposites show enhanced conductivity in the frequency range of 1 to 10^6^ Hz due to the incorporation of noble metal (Pt and Ag) particles in the 3D-graphene nanosheets. The real part of AC conductivity was derived by complex conductivity using the relation:


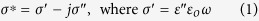


where 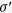 and 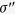 are the real and imaginary parts of complex conductivity.

Figure 5 shows that the conductivity of 3D-graphene increases with increase in the frequency, following the power law. The frequency-dependent total ac conductivity is expressed by the power law:


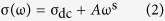


where ω is the angular frequency, exponent s is the function of frequency and is limited to 0.7 to 0.9. σ_dc_ is the frequency-independent conductivity or dc conductivity (at ω=0), and A is the temperature-dependent constant. The addition of noble metal particles in 3D-graphene shows a prominent increase in conductivity, indicating frequency-independent behavior. The AC conductivity (σ′ (ω)) is almost constant over the frequency in 3D-graphene/Ag and 3D-graphene/Pt nanocomposites, which is ascribed to the dc conductivity, in which the electrode polarization is negligible. A similar type of behavior was observed in carbon nanotube-based composites earlier^48^.

The dispersion of noble metal (Pt and Ag) particles on the 3D building blocks of nanosheets increases the conductivity to 0.032 S/cm in 3D-graphene/Ag and 0.01 S/cm in 3D-graphene/Pt nanocomposites. The electrical conductivity of 3D-graphene/noble metal nanocomposites (Ag and Pt) was significant to the compressed graphene nanosheets and graphite oxide reported earlier^43^,^49^. In the present work, the noble metal (Pt and Ag) particles act as bridge between the nanosheets by making coordinate or covalent bonds, and resulting increase in the π-π interaction between the graphene sheets. The homogeneous distribution of noble metals on graphene sheets and presence of large electron clouds in the complexing metals might be increased the electric charge transportation in the 3D-graphene/noble metal nanocomposite monoliths than bare 3D-graphene. However, the conductivity losses were observed in the developed samples due to the porous 3D-graphene nanosheets. Further, the enhanced conductive 3D-graphene/noble metal nanocomposites were extended to EMI studies in which enhanced conductivity is necessary criterion.

**Figure 6:**
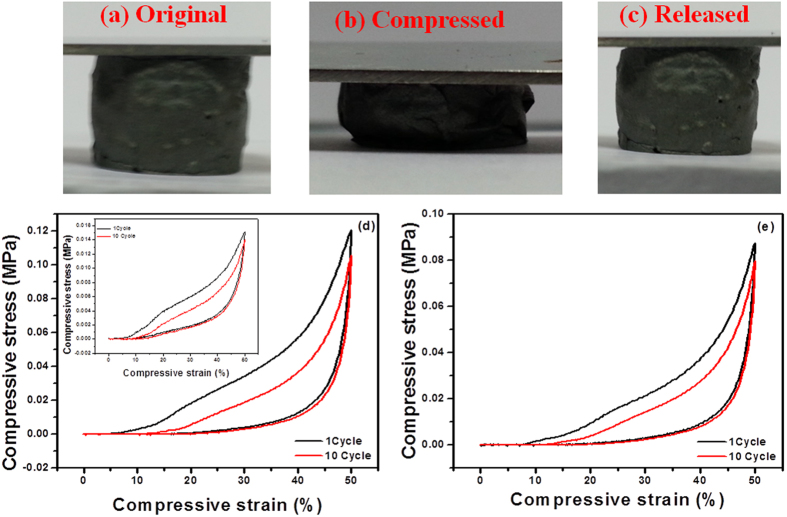
Compressibility of 3D-graphene, 3D-graphene/Pt, and 3D-graphene/Ag naocomposites. (**a**–**c**) Digital images of a typical compression process of 3D-graphene/noble metal nanocomposites. Compressive stress-strain curves of 10 cycles (loading and unloading) of (**d**) 3D-graphene/Pt and (**e**) 3D-graphene/Ag nanocomposites (Inset: Compressive stress–strain curve of 3D-graphene).

The compression test of 3D-graphene/noble metal (Pt and Ag) nanocomposite at 50% strain was investigated to identify the stress-strain behavior under cyclic loading. The 3D-graphene/noble metal (Pt and Ag) nanocomposite was compressed to up to 50% strain under pressure; it recovered its original shape immediately after the pressure was released (Fig. 6(a–c)). The compressive stress-strain curves of the nanocomposite were recorded under cyclic loading up to 10 cycles. The compressive stress-strain curve of bare 3D-graphene (Inset), 3D-graphene/Pt, and 3D-graphene/Ag nanocomposites at 50% strain are shown in the Fig. 6(d,e). In all the cases, the unloading curve almost returned to the initial points after being subjected to fatigue loading of 10 cycles at 50% strain. This suggested that unlike bare 3D-graphene, 3D-graphene/Pt and 3D-graphene/Ag nanocomposites also had excellent superelasticity and complete shape recovery without plastic deformation after release of pressure. The compressive stress decreased to 92% (3D-graphene), 87% (3D-graphene/Pt) and 91% (3D-graphene/Ag) of the original value after 10 compressing cycles, which confirmed good elasticity and flexibility of 3D-graphene/noble metal nanocomposites. A similar type of behavior was observed in carbon-based aerogels earlier^50^,^51^. After, relieving the compressive stress, the morphology study was carried out for the developed samples (Fig. S5, Supporting information). The pores of 3D-graphene were elongated longitudinally under transverse loading after recovery. However, the porous surface of the 3D-graphene/noble metal (Pt and Ag) nanocomposite unchanged without any elongation and cracks, suggesting faster recovery to its initial state (Fig. S5, Supplementary video). The stress-strain curves of the 3D-graphene/Pt composites show linear behavior at ε < 35% with an elastic modulus of 16 kPa, and a nonlinear behavior thereafter with an increasing slope of up to 50% and a modulus of 49 kPa. Similarly, 3D-graphene/Ag exhibits an elastic modulus of 11 kPa in the linear regime and 45 kPa in the nonlinear regime, whereas, 3D-graphene exhibits an elastic modulus of 3 kPa in the linear regime and 5 kPa in the nonlinear regime. The increase in the elastic properties of the 3D-graphene nanocomposites is due to strong force of attraction between the metal nanoparticles and graphene sheets, resulting high load transfer efficiency, and thereby increasing the tenacity of elastic behavior. Similar type of enhanced elastic properties of the nanocomposites was evidenced in the literature earlier^52^. Further, these excellent mechanical properties of 3D-graphene/noble metal nanocomposites can be put forward for various applications in elastic conductors, flexible electrodes and pressure sensors.

### Electromagnetic interference shielding studies

**Figure 7:**
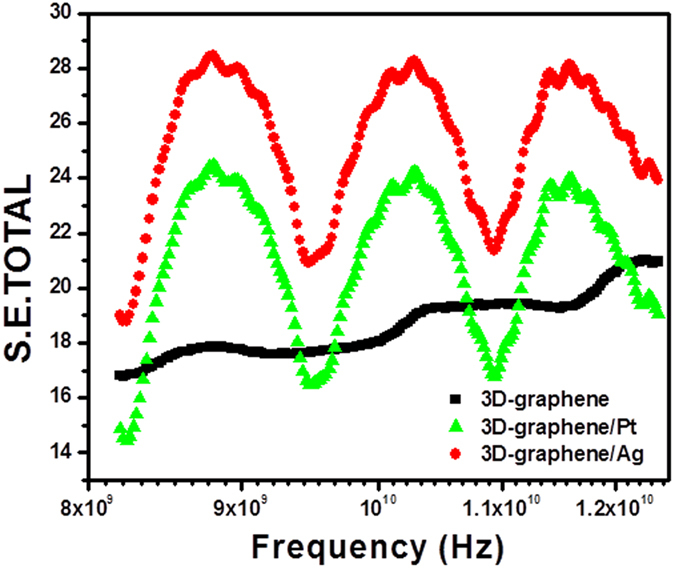
EMI shielding effectiveness (dB) of 3D-graphene, 3D-graphene/Pt, and 3D-graphene/Ag nanocomposites.

In support of conductive studies, the electromagnetic interference shielding properties of 3D-graphene and 3D-graphene/noble metal nanocomposites were studied in the X-band (8.2–12.4 GHz) by measuring shielding parameters using a vector network analyzer (Fig. 7). The shielding effectiveness (SE) was calculated by using the relation^53^


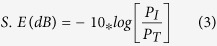


where *P*_*I*_ corresponds to input power, and *P*_*T*_ corresponds to Transmit powers.


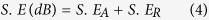



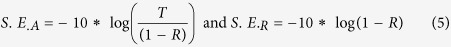


where 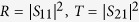


The shielding properties of 3D-graphene/noble metal nanocomposites were compared with the host 3D-graphene. The total SE in the composites is mostly dominated by the absorption phenomenon. It is interesting that the 3D-graphene/Ag nanocomposite showed better shielding properties than 3D-graphene/Pt and 3D-graphene. The SE of 28 dB for the 3D-graphene/Ag nanocomposite put forward a shielding power of ~99.6% in an average film thickness of 0.8 mm. Additionally, 3D-graphene/Pt showed an enhanced SE of 24 dB compared 3D-graphene (~18 dB). The total SE by the absorption phenomenon is mainly due to the interaction of electric dipoles with the electromagnetic field of radiation and the functional groups of functionalized 3D-graphene attained by the addition of noble metal (Pt, Ag) particles. Thus, the incorporation of fillers in the 3D-graphene plays an important role for improving the structure and electrical conductivity, which also revealed by our earlier study^54^. The dispersion of particles on the surface of the 3D building-block nanosheets was uniform and reduced the skin depth by improving the absorption losses, resulting in a higher surface; this further enhances the SE.

SE in terms of permittivity (ε), conductivity (σ′) and permeability (μ′) is expressed as:


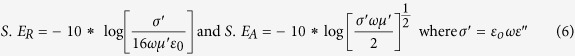


There by suggesting that the proportionate increase in conductivity in the 3D-graphene/Ag nanocomposite leads to substantial enhancement in the shielding effectiveness. Similar results have been reported earlier, in which carbon foam was embedded with Ni particles to improve the shielding properties^55^.

### Electrocatalytic performance

**Figure 8:**
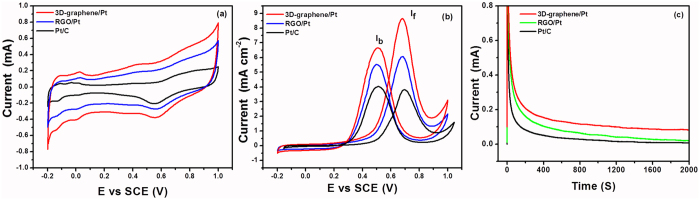
(**a**) CVs of 3D-graphene/Pt, RGO/Pt nanocomposite and Pt/C electrodes in 0.5 M H_2_SO_4_ solution; scan rate: 50 mV s^−1^, (**b**) CVs of 3D-graphene/Pt, RGO/Pt nanocomposite and Pt/C electrodes in 0.5 M H_2_SO_4_ + 1.0 M methanol solution; scan rate: 50 mV s^−1^ and (**c**) Current-time curves of the 3D-graphene/Pt nanocomposite, the RGO/Pt nanocomposite and Pt/C electrodes in a solution of 0.5 M H_2_SO _4_+ 1.0 M methanol at +0.7 V.

In order to explore the potential application of 3D-graphene/noble metal nanocomposites as an electrocatalysts further, we have selected the 3D-graphene/Pt nanocomposite to study its catalytic activity in methanol oxidation. Figure 8(a) presents the cyclic voltammetry curves (CVs) of commercial Pt/C (20 wt%), the RGO/Pt nanocomposite and the 3D-graphene/Pt nanocomposite in the 0.5 M solution of H_2_SO_4_. The CVs are scanned from −0.2 to 1.0 V with a scan rate of 50 mV s^−1^ in the 0.5 M solution of H_2_SO_4_ (N_2_-saturated). It has been observed that there are three potential regions on CVs of the commercial Pt/C (20 wt%), the RGO/Pt nanocomposite and the 3D-graphene/Pt nanocomposite. The first potential region from −0.2 to 0.05 V presents adsorption/desorption of hydrogen. The 2^nd^ potential region from 0.1 to 0.3 V indicates the double layer capacitance region. The 3^rd^ potential region from 0.4 to 0.9 shows the metal oxidation/reduction region. The 3D-graphene/Pt nanocomposite shows higher double–layer capacitance when compared to commercial Pt/C and the RGO/Pt nanocomposite. This is because of the larger surface area of the 3D-graphene as compared to the RGO and carbon black. The larger surface area of 3D-graphene is confirmed by the Brunauer-Emmett-Teller (BET) surface area analysis, which indicates that the specific surface area of the carbon black, RGO and 3D graphene are 94, 122 and 155 m^2^/g (Fig. S6, Supporting information), respectively. The electrochemical active surface area (ECSA) of Pt in the three different catalysts was calculated from the integrated hydrogen adsorption area. It has been observed that the ECSA of Pt in the 3D-graphene/Pt nanocomposite is 20.7 m^2^/g, which is ~ 66% more than that of Pt/C (12.5 m^2^/g) and 36% more than that of the RGO/Pt nanocomposite (15.2 m^2^/g). The higher ESCA of Pt in the 3D-graphene/Pt nanocomposite may be due to the homogenous distribution of the Pt nanoparticles on the 3D-graphene (as indicated in TEM) and the distinct structure of 3D-graphene (Free-standing, interconnected graphene sheets).

The electrochemical activities of the commercial Pt/C (20 wt%), RGO/Pt nanocomposite and the 3D-graphene/Pt nanocomposite were compared by CV for the oxidation of methanol, and the subsequent results are presented in the Fig. 8(b). The peaks at 0.7 V and 0.5 V in the forward and reverse scan correspond to the direct electro-oxidation of methanol adsorbed on the surface of the catalyst, and the oxidation of intermediate carbonaceous residues that are produced on the catalyst surface in the forward scan, respectively^56^,^57^,^58^. Hence, the catalytic performance of the electrocatalysts is generally estimated from the forward scan. Compared to Pt/C and the RGO/Pt nanocomposite, the 3D-graphene/Pt nanocomposite exhibits higher specific current density at 0.7 V. The higher current density suggests that the 3D-graphene/Pt nanocomposite exhibits higher catalytic activity compared to Pt/C (20 wt%) and the RGO/Pt nanocomposite. This is because of the unique structure of 3D-graphene. The 3D structure of graphene promotes mass transfer efficiency of reactants, products, and electrolytes in the reaction process, which increases the electrocatalytic performance of 3D-graphene/Pt nanocomposites. The catalyst’s tolerance of the accumulation of the intermediate carbonaceous species (mostly carbon monoxide) is estimated from the ratio of the forward-scan peak current (I_f_) to the backward-scan peak current (I_b_) that is, I_f_/I_b_ value^59^,^60^. The I_f_/I_b_ value for Pt/C, RGO/Pt, and 3D-graphene/Pt are 0.85, 1.06, and 1.3 respectively, signifying that the 3D-graphene/Pt nanocomposite has higher efficiency in the methanol oxidation in the forward direction.

The long-term catalytic activities of the Pt/C, RGO/Pt nanocomposite and the 3D-graphene/Pt nanocomposite were examined by a steady-state chronoamperometry measurement. Figure 8(c) shows the chronoamperometry data of Pt/C, the RGO/Pt nanocomposite, and the 3D-graphene/Pt nanocomposite at 0.5 V in a solution that contained 1M CH_3_OH and 0.5 H_2_SO_4_ for 2000 s. The initial current decay in all cases is due to the development of double layer capacitance. Subsequently, the current decays slowly, which can be attributed to the adsorption of a small amount of carbon monoxide (CO_ads_) species on the surface of the catalyst during electro-oxidation of methanol^61^. The decay of the current originates from the adsorption of SO_4_^2−^ species on the surface of the catalysts, which inhibits the reaction active sites of the catalysts^62^. The current slowly reaches a quasi-equilibrium steady state after an extended period of operation. Compared to Pt/C and the RGO/Pt nanocomposite, the 3D-graphene/Pt nanocomposite shows a higher steady-state current in methanol electro-oxidation and is in good agreement with the CV result. So, both steady-state current and the CV result illustrate that the 3D-graphene/Pt nanocomposite has better catalytic performance in methanol electro-oxidation.

## Conclusion

In this study, we have demonstrated a general and clean method to fabricate 3D-graphene/noble metal (Pt and Ag) nanocomposites built from partially reduced graphene oxide and metal precursors using a facile and cost-effective freeze-casting method. The as-synthesized 3D-graphene/noble metal (Pt and Ag) nanocomposites consisted of a highly interconnected porous structure and exhibited enhanced mechanical properties and conductivity due to the incorporation noble nanoparticles (Pt and Ag) in the 3D-graphene building block. Their electrocatalytic performance and electromagnetic interference (EMI) shielding effect has been further investigated. The as-synthesized 3D-graphene/noble metal (Pt and Ag) nanocomposites exhibit an excellent EMI shielding effectiveness when compared with bare 3D-graphene, and can be used for electromagnetic radiation or as a radar-absorbing material in civil and military applications. Electrocatalytic activity of the 3D-graphene/Pt nanocomposite in methanol oxidation has been demonstrated, which shows high electrocatalytic activity, reliable stability and abnormally high poison tolerance compared to the RGO/Pt nanocomposite and Pt/C. The resulting 3D-graphene/noble metal (Pt and Ag) nanocomposites with highly interconnected micro and macro porous structures, and good electrical, mechanical and catalytic activities hold considerable promise of a new pathway for application in photocatalysis, pressure sensors, actuators, and electrode materials.

## Additional Information

**How to cite this article**: Sahoo, P. K. *et al.* Ice-templated synthesis of multifunctional three dimensional graphene/noble metal nanocomposites and their mechanical, electrical, catalytic, and electromagnetic shielding properties. *Sci. Rep.* **5**, 17726; doi: 10.1038/srep17726 (2015).

## Supplementary Material

Supplementary Information

Supplementary Video
