# Supplementary material for: Ice-templated synthesis of multifunctional three dimensional graphene/noble metal nanocomposites and their mechanical, electrical, catalytic, and electromagnetic shielding properties
Source: Sci Rep. 2015 Dec 7;5:17726. doi: 10.1038/srep17726 (PMC4671061; doi:10.1038/srep17726)
Supplement: Supplementary Information [file srep17726-s1.doc]

**Supplementary Information**

**Ice-templated synthesis of multifunctional three dimensional graphene/noble metal nanocomposites and their mechanical, electrical, catalytic, and electromagnetic shielding properties**

**P.K. Sahoo^a^, Radhamanohar Aepuru^b^, Himanshu Sekhar Panda** **^b^ and D. Bahadur^a*^**

*^a^ IITB-Monash Research Academy, Indian Institute of Technology Bombay, Mumbai-400076, India.*

*^b^Department of Materials Engineering, Defence Institute of Advanced Technology, Pune-411025, India.*

***Corresponding Authors:**

Prof. Dhirendra Bahadur

Institute Chair Professor

Dept. of Metallurgical Engineering and Materials Science

Indian Institute of Technology Bombay

Powai, Mumbai-400076, India

Ph: 91-22-25767632, Fax: 91-22-25723480

Email: [dhirenb@iitb.ac.in](mailto:dhirenb@iitb.ac.in)

**Figure S1** XRD pattern of the RGO/Pt nanocomposite.

**Figure S2 (A)** Raman spectra of (a) GO, (b) bare 3D-graphene, (c) 3D-graphene/Pt and (d) 3D-graphene/Ag nanocomposites. **(B)** Enlarge G band spectra of (a) GO, (b) bare 3D-graphene, (c) 3D-graphene/Pt and (d) 3D-graphene/Ag nanocomposites.

**Figure S3** FTIR spectra of (a) GO, (b) 3D-graphene/Pt and (c) 3D-graphene/Ag nanocomposites.

**Figure S4** O 1s XPS spectra of GO and 3D-graphene/Pt nanocomposite

**
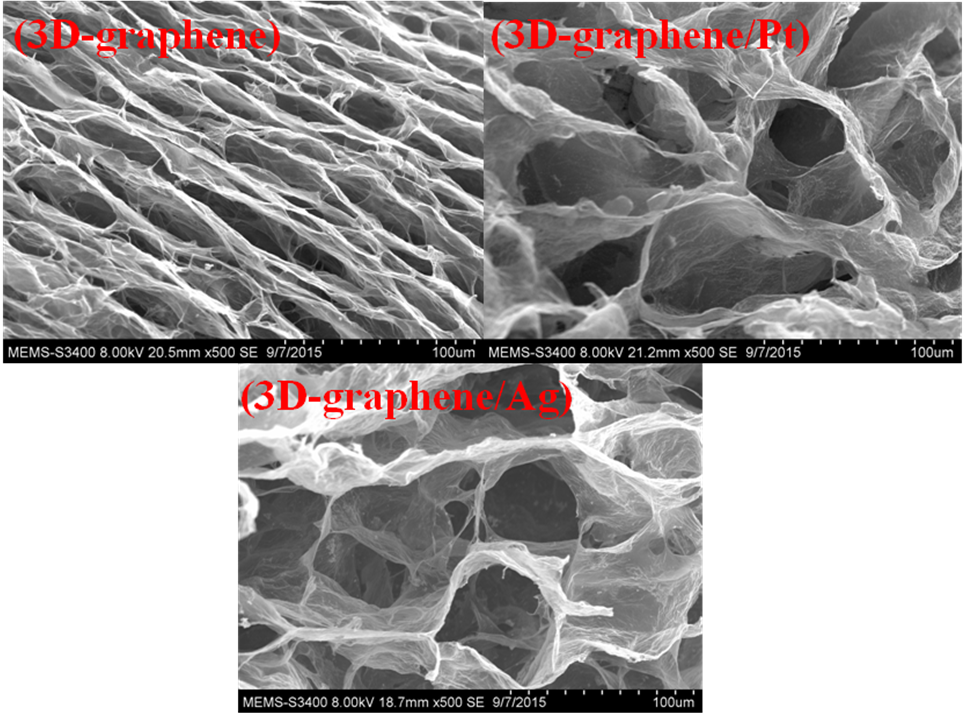
**

**Figure S5** FEG-SEM micrographs of developed materials after application of load.

**Figure S6** N_2_ adsorption-desorption isotherms of (a) Carbon black, (b) RGO and (c) 3D-graphene (d) Meso-Macro pore size distribution of 3D-garphene/Pt nanocomposite
